# Artery to Cystic Duct: A Consistent Branch of Cystic Artery Seen in Laparoscopic Cholecystectomy

**DOI:** 10.1155/2015/847812

**Published:** 2015-07-09

**Authors:** Arshad Rashid, Majid Mushtaque, Rajandeep Singh Bali, Saima Nazir, Suhail Khuroo, Sheikh Ishaq

**Affiliations:** ^1^Department of Health Services, Kashmir 190001, India; ^2^Department of Surgery, Maulana Azad Medical College, New Delhi 110002, India; ^3^Department of Community Medicine, Government Medical College, Srinagar 190010, India; ^4^Department of Surgical Gastroenterology, KEM Hospital, Mumbai 400012, India

## Abstract

Uncontrolled arterial bleeding during laparoscopic cholecystectomy is a serious problem and may increase the risk of bile duct damage. Therefore, accurate identification of the anatomy of the cystic artery is very important. Cystic artery is notoriously known to have a highly variable branching pattern. We reviewed the anatomy of the cystic artery and its branch to cystic duct as seen through the video laparoscope. A single artery to cystic duct with the classical “H-configuration” was demonstrated in 161 (91.47%) patients. This branch may cause troublesome bleeding during laparoscopic dissection in the hepatobiliary triangle. Careful identification of artery to cystic duct is helpful in the proper dissection of Calot's triangle as it reduces the chances of hemorrhage and thus may also be helpful in prevention of extrahepatic biliary radical injuries.

## 1. Introduction

Laparoscopic cholecystectomy is an established gold standard in the treatment of cholelithiasis and is considered to have many advantages over its open counterpart [1]. Though, initially it was associated with a higher incidence of iatrogenic biliary injury and arterial hemorrhage [2–4] perhaps due to a lack of exposure to the laparoscopic anatomy of the gallbladder and proper technical skills. Cystic artery is notoriously known to have a highly variable branching pattern and relation to biliary ducts [5–7]. The reported mortality because of injuries to blood vessels during laparoscopic cholecystectomy is about 0.02% [8] and the conversion rate to open surgery is approximately 0%–1.9% [9]. So it is of utmost importance that the modern laparoscopic surgeon must be aware of the possible arterial and biliary variants in order to minimize surgical mishaps. Many classifications have been published on the branching pattern of cystic artery [10, 11]; however, nothing substantial has been written on the branch of cystic artery that supplies the cystic duct. The current paper attempts to describe the anatomy of this “artery to cystic duct.”

## 2. Materials and Methods

This was a prospective study conducted in two hospitals (a district level and a subdistrict level) of the Department of Health Services, Kashmir, over a period of one year from April 2014 to April 2015 and included 176 consecutive patients of laparoscopic cholecystectomy. Two well-experienced laparoscopic surgeons (first and second author) operated the patients. Laparoscopic cholecystectomy was performed by using Image One high definition camera supplied by Karl Storz, Germany, mounted on a Hopkins II 30° laparoscope. The video was visualized on a high definition wide screen monitor (Karl Storz, Germany). All the procedures were recorded for subsequent review. An approval from the local ethics committee was obtained for this study.

**Figure 1:**
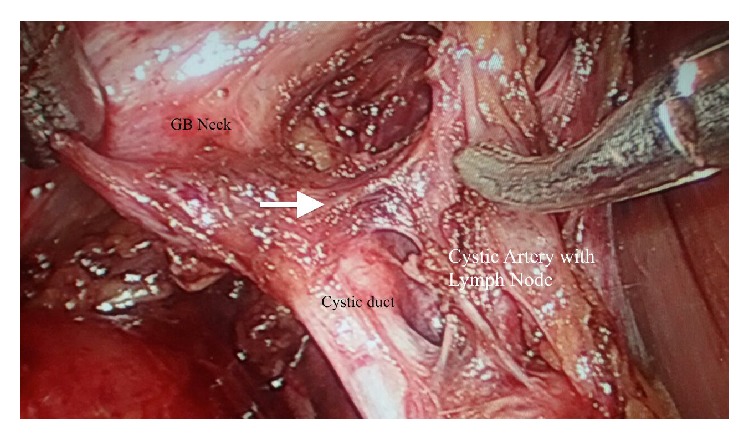
The “*H-configuration*” showing cystic artery, cystic duct, and the artery to cystic duct (white arrow). The GB neck, the CBD, and the cystic Lymph node of Lundt are also shown.

Before surgery, the patients were evaluated with history, physical examination, and baseline investigations like hemogram, liver, kidney function tests, and an abdominal sonogram. The patients/attendants were explained the procedure in detail and a written and informed consent was taken for the procedure. A standard four-port laparoscopic cholecystectomy was performed after creating the pneumoperitoneum by open technique. Meticulous dissection of Calot's triangle was done and the anatomy of cystic duct and cystic artery was delineated properly. The branching pattern of cystic artery was noted with emphasis on the clear delineation of the artery to cystic duct. An attempt was made to demonstrate the “H-configuration” with the vertical limbs of “H” being formed by cystic duct and cystic artery and the connecting limb being formed by artery to cystic duct (Figure 1).

The data thus collected was compiled and analyzed using SPSS version 21 for Mac (IBM Corporation, 2012). The association between variables was calculated for 95% confidence intervals by using “Chi square test.” “Unpaired* t*-test” was used to compare the means. A *P* value < 0.05 was taken as significant.

## 3. Results

Out of 176 patients, 116 were females and 60 were males. The mean age of females was 47.235 ± 3.243 years and that of males was 49.653 ± 2.261 years [*P* value = 0.5329]. The mean operating time was 37.365 ± 10.163 minutes in females and 46.871 ± 11.274 minutes in males [*P* value = 0.0021]. Although body mass index for both genders was comparable (27.232 ± 3.211 in females and 28.991 ± 1.247 in males, *P* value = 0.3765), the average weight was significantly different in women and men (75.131 ± 4.113 kg and 87.525 ± 5.717 kg, resp., *P* value = 0.0039). No conversions were noted in our series.

A classical single cystic artery present within Calot's triangle was seen in 134 (76.14%) patients [89 (76.7%) females and 45 (75%) males, *P* value = 0.9121] and a double cystic artery present within Calot's triangle was noticed in 23 (13.06%) patients [16 (13.8%) females and 7 (11.7%) males, *P* value = 0.0763]. An inferior cystic artery lying outside Calot's triangle inferior to the cystic duct was seen in 19 (10.8%) patients [11 (9.5%) females and 8 (13.3%) males, *P* value = 0.0316].

A single artery to cystic duct with the classical “H-configuration” was demonstrated in 161 (91.47%) patients [109 (93.97%) females and 52 (86.67%) males, *P* value = 0.0023]. Out of 134 patients with classical single cystic artery, we could dissect out the artery to cystic duct in 130 (97.01%) patients [87 (97.75%) females and 43 (95.56%) males, *P* value = 0.6887]. In 23 patients with a double cystic artery, the artery in proximity to the cystic duct supplied a branch to the cystic duct in 21 (91.30%) patients [15 (93.75%) females and 6 (85.71%) males, *P* value = 0.0443], whereas the artery distant from cystic duct did not supply any branch to the cystic duct. We could dissect out the artery to cystic duct with the “H-configuration” in only 10 (52.63%) patients [7 (63.64%) females and 3 (37.5%) males, *P* value < 0.0001] out of 19 patients with an inferior cystic artery. There was a statistically significant difference in the demonstration of artery to cystic duct arising from an inferior cystic artery and a classical single cystic or double cystic artery [*P* value < 0.0001].

## 4. Discussion

The importance of a thorough knowledge of arterial supply of extrahepatic biliary ductal system and its variations lies in the fact that it may help in reducing the uncontrolled bleeding that may increase the risk of intraoperative injuries to vital vascular and biliary structures. The laparoscopic anatomy of the cystic artery can be considered as a precondition for performing safe laparoscopic procedures. Accidents involving vessels or the common bile duct during laparoscopic cholecystectomy can be avoided by careful dissection of Calot's triangle and the hepatoduodenal ligament [12]. In late 20th century, Patil et al. drew attention to the high frequency of variations in the cystic artery and bile ducts in the region of Calot's triangle bordered by the cystic duct, common hepatic duct, and lower edge of the liver [13]. Rocko and Gioia suggested that Calot's triangle should be renamed as the hepatobiliary triangle [14]. Small branches of the cystic artery supplying the cystic duct may cause troublesome bleeding during laparoscopic dissection in the hepatobiliary triangle and were named as Calot's arteries by Rocko and Gioia [14]. In contrast to Hugh's observations, we could only delineate a single arterial branch that supplied the cystic duct.

The cystic artery is a branch of the right hepatic artery and is usually given off in Calot's triangle. It has a variable length and becomes adherent to the gallbladder in the neck or body area. The course and length of the cystic artery in Calot's triangle are variable. Although classically the artery traverses the triangle almost in its center, it can occasionally be very close to or even lower than the cystic duct. It usually gives off an anterior or superficial branch and a posterior or deep branch. In addition, the cystic artery gives off direct branches to the cystic duct [15].

We observed that the artery to cystic duct arises almost at right angles from the cystic artery and enters the cystic duct midway between cystic duct common hepatic duct junction and the cystic duct gallbladder junction to form an “H-configuration.” After this “H-configuration” is demonstrated, we suggest that the artery to cystic duct be cauterized so as to achieve an extra bit of length of the cystic duct, which is especially important when dealing with very short cystic ducts. Cauterizing this artery also helps prevent the troublesome bleeding that may sometimes arise due to an inadvertent injury to it.

The ability to dissect out the artery to cystic duct was significantly lesser in males as compared to females. It appears that owing to more adhesions and thicker peritoneum dissection difficulties in males preclude to lesser demonstration of artery to cystic duct. This was especially seen in patients with classical single cystic artery and an inferior cystic artery.

Cystic artery was divided into three types by Ignjatovic in minimally invasive procedures: type 1 shows normal anatomy; type 2 shows more than one artery in Calot's triangle; and type 3 shows no artery in Calot's triangle [11]. In our study, type 3 arteries were less frequently associated with an artery to cystic duct. Thus, there appears to be a polarity in the branching pattern of cystic artery. Before dividing into two terminal branches, cystic artery preferentially gives off an inferior branch supplying the cystic duct. As was seen in our study, the artery to cystic duct was less frequently found arising from the inferior cystic artery as it had to course superiorly towards cystic duct. There is also a possibility that variant cystic arteries do not give off the artery to cystic duct as consistently as the normal ones. Further research is needed to substantiate these ideas.

We acknowledge the fact that arteriography is the ideal method of delineating the arterial patterns (including artery to cystic duct); however, the current paper focused on the intraoperative laparoscopic anatomy rather than the radiologic anatomy.

## 5. Conclusion

Our study implies that the artery to cystic duct is a very consistent branch of cystic artery and its demonstration is helpful in the proper dissection of Calot's triangle as it reduces the chances of cystic artery hemorrhage and may also be helpful in prevention of extrahepatic biliary radical injuries.
